# Torque efficiency of square and rectangular archwires into 0.018 and 0.022 in. conventional brackets

**DOI:** 10.1186/s40510-016-0118-0

**Published:** 2016-01-15

**Authors:** Spyridon N. Papageorgiou, Iosif Sifakakis, Ioannis Doulis, Theodore Eliades, Christoph Bourauel

**Affiliations:** Department of Orthodontics, School of Dentistry, University of Bonn, Bonn, Germany; Department of Oral Technology, School of Dentistry, University of Bonn, Bonn, Germany; Department of Orthodontics, School of Dentistry, National and Kapodistrian University of Athens, Athens, Greece; Hellenic Air Force General Hospital, Athens, Greece; Clinic of Orthodontics and Paediatric Dentistry, Centre of Dental Medicine, University of Zurich, Plattenstrasse 11, CH-8032 Zurich, Switzerland

**Keywords:** Torque, Moments, 0.018-in. slot, 0.022-in. slot, Steel, Square archwires, Rectangular archwires

## Abstract

**Background:**

The aim of this study was to compare the torque efficacy of square and rectangular wires in 0.018- and 0.022-in. conventionally ligated brackets.

**Methods:**

Brackets of the same prescription were evaluated in both slot dimensions. Identical acrylic resin models of the maxilla were bonded with the brackets and mounted on the Orthodontic Measurement and Simulation System. Ten 0.018 × 0.018 in., 0.018 × 0.022 in., and 0.018 × 0.025 in. stainless steel wires were evaluated in the 0.018-in. brackets and ten 0.019 × 0.019 in., 0.019 × 0.025 in., and 0.019 × 0.026 in. stainless steel wires were evaluated in the 0.022-in. brackets. A 15° buccal root torque was gradually applied to the right central incisor bracket, and the moments were recorded at this position. One-way ANOVA was applied for both bracket slot sizes along with post hoc analysis for the various archwire sizes.

**Results:**

The mean measured moments varied between 10.78 and 30.60 Nmm among the assessed wire-and-bracket combinations. Both square and rectangular archwires in the 0.018-in. bracket system exerted statistically significantly higher moments in comparison with their counterparts in the 0.022-in. bracket system. Rectangular archwires exerted statistically significantly higher moments than square archwires, both for the 0.018- and the 0.022-in. bracket system.

**Conclusions:**

Rectangular archwires seem to be more efficient in torque exertion, especially in 0.018-in. brackets.

## Background

Proper buccolingual inclination of both posterior and anterior teeth is essential to providing stability and proper occlusal relationship in orthodontic treatment. Torque of the maxillary incisors is particularly critical in establishing an esthetic smile line, proper anterior guidance, and a solid Class I relationship, because undertorqued anterior teeth can preclude the retraction of the anterior maxillary dentition. Suboptimal torque of the incisors can deprive the dental arch of space [1], while suboptimal torque of the posterior teeth might not allow appropriate cusp-to-fossa relationships between the maxillary and mandibular teeth [2].

Torque expression is influenced by many factors, including the dimensions and material properties of the archwire and the bracket, the angle of twist of the archwire relative to the brackets, the mode of ligation, the bracket position, irregularities in tooth morphology, and beveling of archwires [3–8]. Slot size is another factor that could potentially influence torque expression. During slide mechanics, 0.022-in. brackets outperform 0.018-in. systems but are inferior in torque expression [9, 10]. With stainless steel archwires of 0.021 in. as the smaller dimension—close enough to the original 0.022-in. bracket slot size to provide full engagement of the bracket slot—springiness and range in torsion are so limited that effective torque with the archwire is essentially impossible. Alternatives that overcome this limitation include the use of nickel-titanium and β-Ti alloys, torquing auxiliaries, or smaller rectangular steel wires, for example, 0.019 × 0.025 in., with increased activations. For this reason, torque prescriptions of the 0.022-in. brackets tend to be exaggerated, since heavy 0.021- or 0.022-in. archwires may never be used in these brackets.

Currently, comparative data on square and rectangular data with regard to the generated moments at the final stages of the treatment is limited. Therefore, the aim of this study was to assess differences in the moments generated in the sagittal plane on a central incisor between square and rectangular stainless steel archwires in 0.018- or 0.022-in. appliances.

## Methods

### Experimental apparatus

**Fig. 1:**
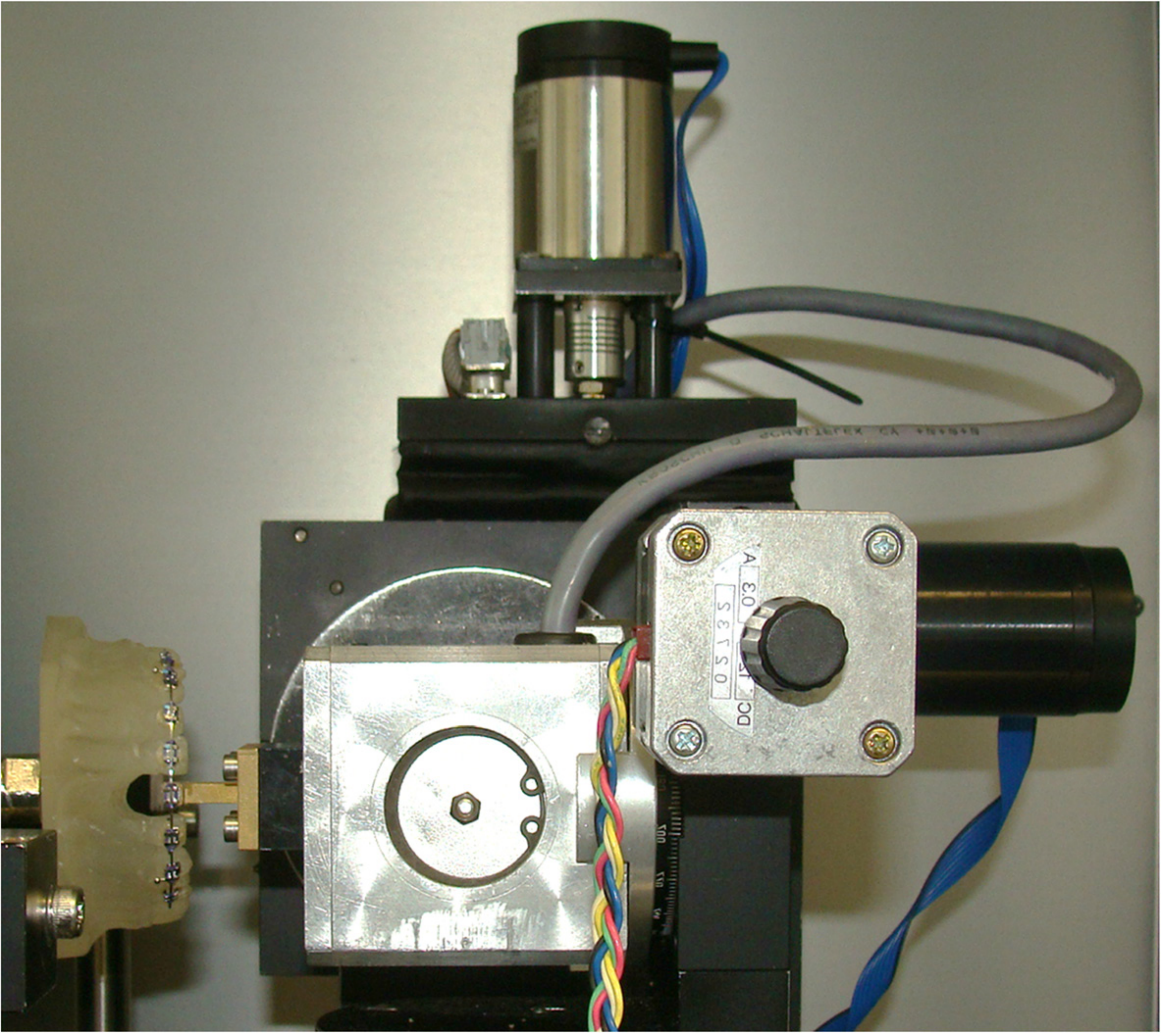
The positioning table of the OMSS with the model mounted on it. Its torque-force sensor replaced the right central incisor

Generated moments (torque) at an upper central incisor was simulated in the Orthodontic Measurement and Simulation System (OMSS) (Fig. 1), a measuring device used widely in the literature for the quantitative evaluation of various orthodontic force systems [11]. Tooth movements can be simulated with this device in the three dimensions [12]. Two independent positioning tables, with six force/torque sensors each, are connected to the region of interest in order to measure the developed force and torque vectors, guided by a central personal computer.

### Configuration and materials

High-torque 0.018- and 0.022-in. brackets from the same company (Mini 2000, ORMCO, Glendora, California, USA) were evaluated with a prescribed torque of 22° and angulation of 5° for the central incisor.

Two identical maxillary models with a leveled and aligned dental arch were constructed from acrylic resin, and each model was bonded with brackets up to the first premolars. An ideal passive 0.018 × 0.025 in. or a 0.021 × 0.025 in. stainless steel archwire was used for bonding the 0.018- and the 0.022-in. brackets, respectively. A torque-force sensor of the OMSS replaced the right central incisor, and the bracket was bonded directly on the sensor. At this configuration, an adjustment of the system was conducted with the abovementioned archwire in place and all forces/moments generated were nullified.

Ten specimens of 0.018 × 0.018 in., 0.018 × 0.022 in., and 0.018 × 0.025 in. stainless steel archwires (ORMCO, Glendora, California, USA) were evaluated in the 0.018-in. brackets. In the 0.022-in. series, the measured archwires were ten 0.019 × 0.019 in., ten 0.019 × 0.025 in., and ten 0.019 × 0.026 in. stainless steel specimens (ORMCO, Glendora, California, USA). For the construction of all archwires, a photocopy of the model was used as a template. The archwires were ligated with 0.120-in. (Molded “O”; ORMCO, Orange, California, USA) elastomeric ligatures into the brackets. A 15° buccal root torque was gradually applied to the right central incisor bracket, in steps of 0.5° along the central axis of the slot. After each activation, the bracket was set to its initial position and the moments in the sagittal plane were recorded during the rotation of the bracket.

After ligating each wire with new elastomerics, the measurement was repeated. The measuring range of the torquing moments in OMSS was ±450 Nmm, and the torque threshold was 0.2 Nmm. The OMSS during the measurement cycles was installed in a temperature-controlled chamber (VEM 03/400, Vötsch Heraeus, Germany) [11].

### Statistical analysis

The mean value of the two repeated measurements in every specimen of the generated moments was calculated at the maximum rotation. We conducted one-way analysis of variance (one-way ANOVA) with the mean torque as the dependent variable and archwire size as the independent/factor variable. One model was fitted for bracket slot 0.018 in. and one for 0.022 in. The three levels for the archwire size for the first model were 0.018 × 0.018 in., 0.018 × 0.022 in., and 0.018 × 0.025 in., while for the second model they were 0.019 × 0.019 in., 0.019 × 0.025 in. and 0.019 × 0.026 in. *Post hoc* analysis followed as multiple comparisons corrected with Sidak’s method. Finally, we executed ANOVA diagnostics to test for the validity of all underlying model assumptions. The alpha level of statistical significance was set to *α* = 0.05. All statistical analyses were performed with the Stata 13 statistical software (Stata Corp, College Station, Texas, USA).

## Results

In the 0.018-in. brackets, the mean maximum moment recorded at 15° in the central incisor by the square 0.018 × 0.018 in. archwire was 18.19 Nmm (SD = 0.30). In the same configuration, but with a rectangular 0.018 × 0.022 in. or a 0.018 × 0.025 in. archwire, the measured mean moment was 22.93 Nmm (SD = 0.68) and 30.60 Nmm (SD = 0.37), respectively (Table 1).

**Table 1:** Mean values, standard deviation (SD) of moments (Nmm) by type of bracket and wire

Bracket slot height (in.)	Cross-section	Wire (in.)	Moment mean (SD)
0.018	Square	0.018 × 0.018	18.19 (0.30)
	Rectangular	0.018 × 0.022	22.93 (0.68)
	Rectangular	0.018 × 0.025	30.60 (0.37)
0.022	Square	0.019 × 0.019	10.78 (0.86)
	Rectangular	0.019 × 0.025	15.66 (0.52)
	Rectangular	0.019 × 0.026	16.51 (0.48)

*SD* standard deviation

In the 0.022-in. brackets, the insertion of a square 0.019 × 0.019 in. archwire generated mean moments of 10.78 Nmm in the central incisor (SD = 0.86). The insertion of a rectangular 0.019 × 0.025 in. or a 0.019 × 0.026 in. archwire exerted a measured mean moment of 15.66 Nmm (SD = 0.52) and 16.51 Nmm (SD = 0.48), respectively. One-way ANOVA rejected the null hypotheses that mean torque was equal for the three archwire sizes, a finding concerning both bracket slot sizes 0.018 in. and 0.022 in. (Tables 2, 3, 4, and 5). The ANOVA results are shown at Tables 2 and 4. *Post hoc* analyses showed that torque exhibited a statistically significant increase by increasing archwire size for both bracket slot dimensions. Tukey’s post hoc analyses are displayed at Tables 3 and 5. Normality and homoscedasticity assumptions were not violated.

**Table 2:** ANOVA results for the effect of wire type on the generated moments on the central incisor for bracket slot size 0.018 in.

Number of observations	30		*R*-squared	0.9921	
Root MSE	0.48		Adj *R*-squared	0.9915	
Source	Partial SS	Df	MS	*F*	Prob > *F*
Model	784.50	2	392.25	1689.13	<0.001
Wire	784.50	2	392.25	1689.13	<0.001
Residual	6.27	27	0.23		
Total	790.77	29	27.27		

*ANOVA* analysis of variance, *MSE* mean square of the error, *SS* sum of squares, *Df* degrees of freedom, *MS* mean square

**Table 3:** Tukey’s post hoc analysis for all pairwise comparisons among archwire sizes for bracket slot size 0.018 in.

Comparison of wires (in.)	Mean difference	HSD statistic	*p* value	Tukey’s 95 % CI
0.018 × 0.018 vs 0.018 × 0.022	4.73	31.07	<0.001	[4.20 , 5.27]
0.018 × 0.018 vs 0.018 × 0.025	12.41	81.44	<0.001	[11.88 , 12.94]
0.018 × 0.022 vs 0.018 × 0.025	7.68	50.37	<0.001	[7.14 , 8.21]

*HSD* honest significant difference, *CI* confidence interval

**Table 4:** ANOVA results for the effect of wire type on the generated moments on the central incisor for bracket slot size 0.022 in.

Number of observations	30		*R*-squared	0.9446	
Root MSE	0.65		Adj *R*-squared	0.9405	
Source	Partial SS	Df	MS	*F*	Prob > *F*
Model	191.42	2	95.71	230.05	<0.001
Wire	191.42	2	95.71	230.05	<0.001
Residual	11.23	27	0.42		
Total	202.65	29	6.99		

*ANOVA* analysis of variance, *MSE* mean square of the error, *SS* sum of squares, *Df* degrees of freedom, *MS* mean square

**Table 5:** Tukey’s post hoc analysis for all pairwise comparisons among archwire sizes for bracket slot size 0.022 in.

Comparison	Mean difference	HSD statistic	*p* value	Tukey’s 95 % CI
0.019 × 0.019 vs 0.019 × 0.025	4.88	23.91	<0.001	[4.16 , 5.59]
0.019 × 0.019 vs 0.019 × 0.026	5.74	28.12	<0.001	[5.02 , 6.45]
0.019 × 0.025 vs 0.019 × 0.026	0.86	4.21	<0.001	[0.14 , 1.58]

*HSD* honest significant difference, *CI* confidence interval

## Discussion

The aim of this study was to assess the torque efficiency of square and rectangular stainless steel archwires in 0.018- and 0.022-in. brackets. The results indicate that high-dimensional rectangular archwires exert significantly higher moments compared to square archwires. Additionally, 0.018-in. brackets were more torque-efficient than 0.022-in. brackets, regardless of archwire cross-section.

Rectangular archwires generated higher moments compared to square ones, both in the 0.018- and the 0.022-in. bracket slot system. This is in accordance with theoretical trigonometrical calculations of the torque play based on their nominal dimensions (Table 6) [13] and with experimental measurements of the actual torque play [3, 14, 15].

**Table 6:** Theoretical torque loss for different square and rectangular archwires in 0.018- and 0.022-in. bracket slots (all with nominal dimensions—based on Dellinger [13])

Wire cross-section	Wire size (in.)	Slot size (in.)	Calculated torque loss (degrees)
Square/rectangular	0.018 × 0.018/0.022/0.025	0.018	0
Square	0.019 × 0.019	0.022	9.96
Rectangular	0.019 × 0.022	0.022	8.37
Rectangular	0.019 × 0.025	0.022	7.24

Torque efficiency was significantly higher with 0.018-in. slot brackets than with 0.022-in. brackets, independently of the archwire’s cross-section. The maximum torque exerted from the 0.019 × 0.025 in. archwire in the 0.022-in. brackets was about half of the value recorded from the 0.018 × 0.025 in. archwire in the 0.018-in. brackets. Between the evaluated square archwires, the magnitude of the recorded moment with a 0.018 × 0.018 in. archwire in 0.018-in. brackets was almost double in comparison with a 0.019 × 0.019 in. archwire in the 0.022-in. brackets. This fact may be explained by the lower torsional play of the final archwires used in the 0.018-in. slot brackets and agrees with previous data [16]. The difference between the 0.019 × 0.025 in. and 0.019 × 0.026 in. archwires in the 0.022-in. brackets was small (5 %) and clinically insignificant. The difference between these archwires in polar molar of inertia and polar section modulus, which are proportional to stiffness and strength in torsion, respectively, is also small (7 %) [17].

According to Burstone, clinically relevant torque values range between 5 and 20 Nmm, with no tooth movement occurring under 5 Nmm, and values exceeding 20 Nmm being associated with damage to the periodontal tissues and particularly root resorption [18]. The time of treatment with rectangular archwires contributes significantly to apical root resorption [19], and teeth that are moved for a longer time or with a higher magnitude of applied moments tend to show a higher degree of root resorption in width and depth [20]. Surprisingly, lower moment magnitudes were found to induce root resorption, too [21]. Root resorption is a multifactorial phenomenon with complex etiopathology, and no single mechanical factor like root torque can adequately cover this. Additionally, deformation of the periodontal ligament and the subsequently developed strains are theoretically influenced by the center of rotation and its relation to its center of resistance, which might not be constant, due to the varying degree of periodontal anisotropy [22]. A changing center of rotation during orthodontic movement is the rule rather than the exception; that is, different types of orthodontic movement might be involved in the movement path [23]. An additional detrimental factor for the development of root resorption might be the iatrogenous approximation of anterior tooth roots towards the palatal cortical plate [24, 25]. Additionally, torque values higher than 26 Nmm have also been associated with plastic deformation of the bracket slot [26]. As a result, the present findings could be used for comparison purposes, but should not be regarded as the sole influencing factors on the ideal torquing efficiency of the various wire-bracket configurations.

In this study, stainless steel archwires were included, as the primary aim was to compare the torque efficiency between square and rectangular archwires. Stainless steel archwires generate higher moments compared with their β-Ti counterparts, in both slot systems [14, 27].

The wires evaluated in this study are most usually inserted as the final archwires during orthodontic treatment, and heavier archwires are rarely used [8]. In both bracket slot sizes, the measured moments generated by rectangular archwires were higher compared to the square archwires, due to torque loss. The torsional play of a 0.018 × 0.025 in. archwire with nominal dimensions in 0.018-in. systems could be theoretically estimated at approximately 0° and at 7° for a 0.019 × 0.025 in. archwire in the 0.022-in. slot [13, 17, 28, 29]. However, various experimental configurations revealed that torque play is actually higher than calculated, both in conventional [30–34] and self-ligating bracket systems [35–39]. The inconsistency in torque play assessments between theoretical calculations and experimental configurations could be attributed to dimensional inconsistency of archwire and bracket, as well as to rounded wire edges [29, 32, 40, 41]. In addition, the bracket slot could be tapered slightly, resulting in further torque-loss fluctuations between archwires of different cross-sections [29]. As the OMSS configuration approximates the clinical situation, the torque loss is notably higher than in the in vitro activating experiments. This is due to additional torque play provided by the adjacent teeth [30], that is, the play both in the torque-receiving and in the torque-delivering bracket must be negated [42].

In the present study, the influence of the varying interbracket wire length [43] is negligible, as models and brackets among the assessed wire-bracket combinations were identical. Stiffness in torsion is inversely proportional to length; however, changes in wire length do not exert as high an influence on wire torsion as on wire bending [17].

Both wire types in this experiment were ligated with elastic ligatures. The effect of elastic/metal ligation type is not expected to influence torque magnitude in full slot size wires and in the 0.018 × 0.025 in. steel archwire in the 0.018-in. slot system. However, for the 0.019 × 0.025 in. steel wire in the 0.022-in. slot, the measured moment with elastic ligation could be 20 % lower than with metal ligation at 5°–15° of torque, since the archwire may not completely seat during torquing [44]. The 0.120-in. elastic ligatures presenting high seating force were used in this experiment in order to ensure the initial seating of the archwire with consistent and similar ligation forces between the different bracket systems [45, 46]. Unfortunately, the main disadvantage of the elastic ligatures still remains their rapid force loss—which could exceed 50 % in 24 h—and consequently, this fact makes the engagement of the wire into the slot flexible and incomplete. In cases of maximum torque demands, steel ligatures should be preferred to provide increased torque expression [2, 45].

This study’s results, as is with most in vitro studies, might not be directly extrapolated to clinical practice. This study has focused on the comparison of the initial force systems of specific bracket/archwire combinations, but the actual force system acting on the teeth will probably vary in time, due to the anisotropic periodontal ligament. Although OMSS can precisely simulate the initial tooth movement within the periodontium, additional factors like intraoral ageing of fixed appliances and the modifying role of saliva are not taken into account.

## Conclusions

Square archwires produce lower torque magnitudes in comparison with rectangular archwires. This difference is exaggerated with a 0.018-in. bracket slot system, in comparison with a 0.022-in. slot system.

The most efficient archwire-bracket combination in terms of torque expression is the use of rectangular archwires in 0.018-in. brackets.
